# Atypical posterior reversible encephalopathy syndrome associated with Lenvatinib therapy in a patient with metastatic thyroid cancer—A case report

**DOI:** 10.1002/cnr2.1605

**Published:** 2022-03-03

**Authors:** Mahajan Abhishek, Ashtekar Renuka, Agarwal Ujjwal, Choudhari Amit, Patil Vijay, Noronha Vanita, Menon Nandini, Prabash Kumar

**Affiliations:** ^1^ Department of Radiodiagnosis and Imaging Tata Memorial Hospital, Tata Memorial Centre, Homi Bhabha National Institute Mumbai Maharashtra India; ^2^ Department of Medical Oncology Tata Memorial Hospital, Tata Memorial Centre, Homi Bhabha National Institute Mumbai Maharashtra India

**Keywords:** drug‐induced posterior reversible encephalopathy syndromeradioimaging, head and neck cancer, immunotherapy, Lenvatinib

## Abstract

Posterior reversible encephalopathy syndrome (PRES) is a disorder of reversible subcortical vasogenic brain oedema in patients with acute neurological symptoms. Drug‐induced PRES has been described with the usage of drugs that target receptors regulating vascular permeability or altering immune response. Lenvatinib is a receptor tyrosine kinase inhibitor that inhibits the kinase activities of vascular endothelial growth factor receptors implicated in cancer progression in addition to their normal cellular functions. The oedema associated with PRES is a consequence of disruption of cerebral blood flow autoregulation. Herein, we present a case of a 77‐year‐old lady who was on treatment with Lenvatinib for metastatic thyroid cancer who subsequently developed PRES. Her clinical and radiological findings improved after discontinuing Lenvatinib and the patient was switched to a different drug and remains asymptomatic on the same. This is the first such report of atypical findings of PRES in a patient on Lenvatinib therapy. Recognition of this entity is crucial for timely withdrawal of the drug and prevent further morbidity and mortality.

## 1 INTRODUCTION

Posterior reversible encephalopathy Syndrome (PRES) is a disorder of reversible subcortical vasogenic brain oedema in patients with acute neurological symptoms. Drug‐induced PRES has been described with the usage of drugs that target receptors regulating vascular permeability or altering immune response. Lenvatinib is a receptor tyrosine kinase (RTK) inhibitor that inhibits the kinase activities of vascular endothelial growth factor (VEGF) receptors implicated in cancer progression in addition to their normal cellular functions. The oedema associated with PRES is a consequence of disruption of cerebral blood flow autoregulation. A 77‐year‐old lady with known history of hypertension and on Lenvatinib for treatment of metastatic thyroid cancer presented with raised blood pressure and left arm weakness. MRI of the brain revealed T2/FLAIR hyperintense signals in bilateral centrum semiovale, periventricular white matter predominantly along the posterior horns, bilateral thalamus, midbrain and pons. A diagnosis of atypical PRES associated with Lenvatinib was made with subsequent discontinuation of the drug. This is the first such case report of atypical PRES associated with Lenvatinib use. This patient also had Lenvatinib associated nephrotic syndrome.

## 2 CASE HISTORY

A 77‐year‐old lady with known hypertension on treatment for the past 15 years presented to our hospital in November, 2020 with history of weight loss of over 20 kg over 6 months, throat pain and right sided neck swelling since July, 2020. Whole body (positron emission tomography) PET‐CECT was done for evaluation of primary disease and possible distant metastases, which showed a large solid metabolically active thyroid nodule in the right lobe, measuring 9.5 × 6.0 × 9.7 cm displacing bilateral carotid and jugular vessels and extending up to the hyoid bone, causing marginal tracheal luminal compression. Another FDG‐avid lesion was seen in segment VIII of the liver, which following fine needle aspiration cytology was proven to be metastasis from papillary thyroid cancer. After consultation in the head and neck multidisciplinary clinic, the disease was deemed unresectable and the patient was started on chemotherapy with docetaxel, cisplatin and 5‐fluorouracil (DCF) regimen. However, it was discontinued after the first cycle in view of toxicity requiring intensive care unit (ICU) admission. The patient subsequently received nine cycles of paclitaxel and carboplatin from 25/8/2020 till 24/10/2020. Response scan showed no significant change in the size of the primary lesion along with new onset metabolically active mediastinal nodes, bilateral pulmonary nodules, stable liver lesion in segment VIII and tumour thrombus in the right internal jugular vein, suggesting disease progression. Her performance status was two, but the patient was relatively asymptomatic for the disease.

**FIGURE 1:**
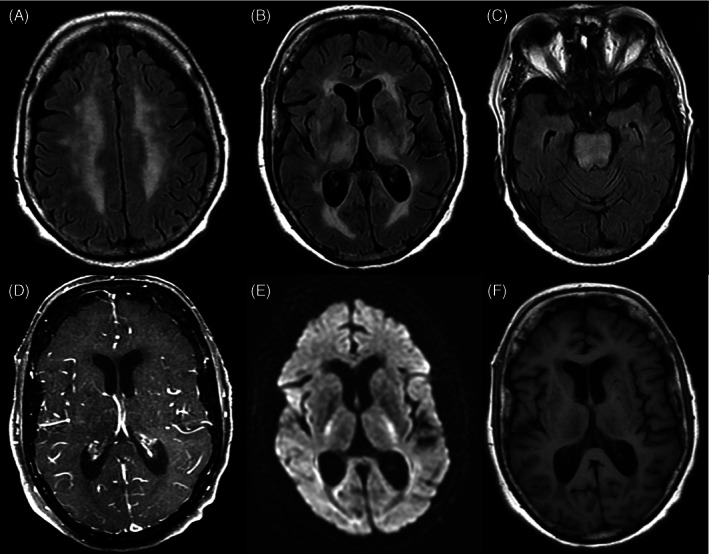
Multiplanar brain MR images of 77‐years old female on Lenvatinib for treatment of metastatic thyroid cancer, who presented with left arm weakness. Upper panel of Fluid‐attenuated inversion recovery (FLAIR) sequence images show hyperintense signal in the centrum semiovale and corona radiata (A), periventricular white matter (B), and brainstem (C). There was no significant post‐contrast enhancement (D), restriction on diffusion weighted images (E) or associated T1 abnormality (F). The T2 hyperintensity along the in the brain‐stem extending along the basal ganglia into the corona‐radiata and centrum semiovale with the given clinical history and treatment details were diagnostic of atypical PRES, likely Lenvatinib induced

A repeat biopsy from the thyroid mass along with molecular and next generation sequencing (NGS) was advised. It revealed features consistent with follicular neoplasm of thyroid, favouring carcinoma. A possibility of poorly differentiated thyroid carcinoma could not be excluded. On immunohistochemistry, tumour cells expressed TTF1 and focally expressed HBME‐1. Ki‐67 (Mib1) labelling index was 10%–12%. NGS report showed Tier I (Pathogenic) missense mutation in exon 9 of PIK3CA gene, Tier I (Pathogenic) missense mutation in exon 3 of NRAS gene and Tier III (variant of unknown significance) missense mutation in exon 1 of MYOD1 gene. After discussion in a molecular tumour board, patient was started on Lenvatinib (10 mg/day) with palliative intent. Patient was monitored for toxicity for the first week after initiating therapy and dose was escalated to 14 mg/day. Patient tolerated the treatment well for the next 3 months with clinical improvement in symptoms and decrease in size of the thyroid mass and liver lesion in the response PET‐CT. However, it was still deemed unresectable in view of extensive locoregional disease. Lenvatinib therapy was continued further in view of clinico‐radiological benefit. Four months after initiating Lenvatinib, patient had an episode of hypertensive emergency, with blood pressure of 200/120 mm Hg and acute onset severe headache and left arm weakness. MRI revealed a chronic lacunar infarct in the right posterior part of pons with T2‐weighted and fluid‐attenuated inversion recovery (FLAIR) hyperintense signals in bilateral centrum semiovale, periventricular white mater predominantly along the posterior horns, bilateral thalamus, midbrain and pons‐ suggestive of atypical PRES.(Figure 1).

The patient was admitted for further management, and following discontinuation of Lenvatinib and administration of steroids there was improvement in the patient's symptoms. The left arm weakness resolved completely within 1 week of discontinuation. Patient had persistent imbalance while walking for over 2 weeks, which at the time of writing has completely resolved. In accordance with the LENVIMA guidebook, which contains information about the proper use of Lenvatinib, occurrence of a grade 4 event such as PRES warranted discontinuation of the drug. Associated with this patient has proteinuria (24‐hour proteinuria 3376 mg/24 h), hypoalbuminemia (2.4 g/dl), low haemoglobin (7.0 g/dl), hypothyroidism. The serum sodium and creatinine were 128 mmol/L and 1.29 mg/dl respectively. The patient was diagnosed to have Lenvatinib induced Nephrotic syndrome. The final diagnosis was Lenvatinib associated grade 4 toxicity (Atypical PRES [involving brainstem] and Nephrotic syndrome). Considering the risk of intracranial/intraparenchymal bleed, acute renal failure and chronic renal failure, Lenvatinib was discontinued. At the time of writing, patient is on treatment with steroids for nephrotic syndrome and Pazopanib for thyroid cancer, which she is tolerating well. The patient has no residual neurological deficits suggesting complete reversal of changes seen with PRES.

## 3 DISCUSSION

This is the first case report describing atypical PRES with Lenvatinib use in a case of metastatic thyroid cancer. PRES, also referred to as reversible posterior leukoencephalopathy syndrome (RPLS) is a disorder of reversible subcortical vasogenic brain oedema in patients with acute neurological symptoms (e.g., seizures, encephalopathy, headache, and visual disturbances) in the setting of renal failure, blood pressure fluctuations, cytotoxic drugs, autoimmune disorders, and pre‐eclampsia or eclampsia.^1^ The oedema associated with PRES is a consequence of disruption of cerebral blood flow autoregulation. The balance of vasoconstriction and vasodilation in response to changing cerebral perfusion pressures works to keep the cerebral blood flow fairly constant. It is thought that when this autoregulatory mechanism breaks down, either in response to rapid changes in cerebral perfusion pressure or a systemic inflammatory response, intravascular fluid leaks out causing cerebral oedema that ultimately leads to PRES.^2^

Three radiologic patterns of PRES have been described depending on the location and extent of white‐matter involvement, usually resulting in hyper‐intense signals on T2‐weighted and FLAIR sequences in a brain MRI. The parieto‐occipital pattern involves the posterior brain, and is the origin of the acronym for PRES. A hemispheric watershed pattern has also been described in the literature, with involvement of the border zones between the anterior cerebral artery and the middle cerebral artery, thus manifesting in oedematous changes to the frontal, parietal and occipital lobes. In atypical cases involvement of other regions has been described, including the cerebellum and brainstem, but these are usually associated with parieto‐occipital changes.^1^ A study analysing imaging patterns of PRES involvement in 136 patients, demonstrated that atypical lesions were not infrequent‐ 14% in basal ganglia, 13% in brain stem and 18% in deep white matter. Recognising these “atypical” locations‐ as seen in our case‐ as part of PRES can be important, particularly if traditional expressions of patterns are incomplete.^3^ PRES is generally reversible, both radiographically and clinically, and has a favourable prognosis.

Drug‐induced PRES has been described with the usage of drugs that target receptors regulating vascular permeability or altering immune response.^4^ Lenvatinib is a RTK inhibitor that inhibits the kinase activities of VEGF receptors implicated in pathogenic angiogenesis, tumour growth, and cancer progression in addition to their normal cellular functions.^5^ TKI‐associated PRES, while rare, is emerging as a serious adverse event related to treatment. Multiple case reports have described PRES associated with use of these agents. Interference with the VEGF pathway is suspected to be a common theme in the pathophysiology. According to the LENVIMA guidebook, across clinical studies of 1823 patients who received LENVIMA as a single agent reversible posterior leukoencephalopathy syndrome (RPLS) occurred in 0.3%.^6^ One study suggested that severe hypertension causing vasogenic oedema can also precipitate PRES.^7^ The LENVIMA guidebook reported that 70% of patients treated with Lenvatinib developed hypertension in a phase III trial.^5^ Thus, it is possible that exacerbation of hypertension related to Lenvatinib use contributed to the appearance of PRES in this patient who had been previously well‐controlled on antihypertensive medication.

Treatment of PRES is largely supportive and symptom targeted. For those cases presenting with elevated blood pressure, reduction of the mean arterial pressure by 25% in the first several hours are usually suggested. In such cases where drug‐induced PRES is suspected, stopping the offending drug is recommended. Complications can include localised ischemia of involved brain regions, as well as intracranial haemorrhage, and frequent neurological monitoring is paramount.

In conclusion, this is the first report about a case of atypical PRES induced by Lenvatinib treatment for metastatic thyroid cancer. Symptoms and radiological features of PRES were seen after 4 months of continuous treatment, with clinical improvement of symptoms following discontinuation of Lenvatinib. Early recognition of this entity is crucial to avoid serious complications arising from continued usage.

## CONFLICT OF INTEREST

The authors declared that they have no conflict of interest to this work.

## AUTHOR CONTRIBUTIONS

**Mahajan Abhishek:** Conceptualization (equal); supervision (equal); validation (equal); visualization (equal); writing – original draft (equal). **Ashtekar Renuka:** Validation (equal); visualization (equal); writing – original draft (equal); writing – review and editing (equal). **Agarwal Ujjwal:** Supervision (equal); visualization (equal); writing – review and editing (equal). **Choudhari Amit:** Resources (equal); supervision (equal); validation (equal); visualization (equal). **Patil Vijay:** Resources (equal); supervision (equal); validation (equal). **Noronha Vanita:** Resources (equal); supervision (equal); validation (equal); visualization (equal). **Menon Nandini:** Supervision (equal); validation (equal); visualization (equal); writing – review and editing (equal). **Prabash Kumar:** Project administration (equal); supervision (equal); validation (equal); visualization (equal); writing – review and editing (equal).

## ETHICS STATEMENT

Informed consent was obtained from the patient.

## Data Availability

Na.
